# A Novel Porous Gelatin Composite Containing Naringin for Bone Repair

**DOI:** 10.1155/2013/283941

**Published:** 2013-02-04

**Authors:** Kuo-Yu Chen, Kuen-Cherng Lin, Yueh-Sheng Chen, Chun-Hsu Yao

**Affiliations:** ^1^Department of Chemical and Materials Engineering, National Yunlin University of Science and Technology, Yunlin 64002, Taiwan; ^2^School of Chinese Medicine, China Medical University, 91 Hsueh-Shih Road, Taichung 40402, Taiwan; ^3^Department of Biomedical Imaging and Radiological Science, China Medical University, 91 Hsueh-Shih Road, Taichung 40402, Taiwan

## Abstract

As Gu-Sui-Bu (GSB) is a commonly used Chinese medical herb for therapeutic treatment of bone-related diseases, naringin is its main active component. This study elucidates how various concentrations of naringin solution affect the activities of bone cells, based on colorimetric, alkaline phosphatase activity, nodule formation, and tartrate-resistant acid phosphatase activity assays to determine the optimal concentration of naringin. GGT composite was obtained by combining genipin cross-linked gelatin and **β**-tricalcium phosphate. GGTN composite was prepared by mixing GGT composite with the predetermined concentration of naringin. Porous GGT and GGTN composites were then made using a salt-leaching procedure. The potential of the composites in repairing bone defects was evaluated and compared *in vivo* by using the biological response of rabbit calvarial bone to these composites. Consequently, the most effective concentration of naringin was 10 mg/mL, which significantly enhanced the proliferation of osteoblasts, osteoclast activity, and nodule formation without affecting the alkaline phosphatase activity of osteoblasts and mitochondrial activity of mixed-bone cells. Radiographic analysis revealed greater new bone ingrowth in the GGTN composite than in the GGT composite at the same implantation time. Therefore, the GGTN composite is highly promising for use as a bone graft material.

## 1. Introduction

An ideal bone replacement material must be biocompatible, biodegradable, and osteoconductive. Numerous materials have received considerable attention as bone substitutes to repair various shapes and sizes of bone defects caused by trauma, infections, tumor resections, or skeletal abnormalities. Tricalcium phosphate (Ca_3_(PO_4_)_2_), a synthetic bone-promoting biomaterial, is commonly used to improve the osteoconductive characteristics of bone replacements [1]. However, remaining within the reconstructed area is rather difficult. Gelatin, a derivative of collagen, is a biocompatible and biodegradable biomaterial that can hold the tricalcium phosphate in place.

Our previous study developed a biodegradable and biocompatible composite as a bone substitute, which consists of gelatin/tricalcium phosphate mixture cross-linked with genipin, a natural cross-linking reagent extracted from the fruits of *Gardenia jasminoides *Ellis. It showed that the gelatin molecular and calcium ions gradually released from the composite enhances the proliferation and differentiation of the osteoblasts [2]. Additionally, *in vivo* studies demonstrated that the composite is biocompatible and osteoconductive [3, 4]. Recent studies have attempted to improve the osteoinductive characteristics of bone substitutes by introducing bone growth factors [5]. Although gelatin-based matrix can serve as a biodegradation carrier for osteoinductive agents and bone growth factors to promote bone healing [6], protein growth factors are relatively expensive.

Our recent studies have evaluated the feasibility of integrating traditional Chinese medicine with gelatin-based composites as bone substitution materials [7–10]. As is widely assumed, Chinese medicine has therapeutic effects on bone healing with an appropriate biodegradation delivery system. The traditional Chinese medicine Gu-Sui-Bu (*Drynaria fortunei* (Kunze) J. Sm) is widely administered in treating bone-related diseases including osteoporosis, bone fracture, and arthritis [11]. Many studies have demonstrated that Gu-Sui-Bu extract (GSB) can increase the proliferation and differentiation of various osteoblastic cells including rat osteosarcoma cells (UMR-106), mouse osteoblastic cells (MC3T3-E1), and primary rat osteoblastic cells [12–16]. Moreover, GSB has positive therapeutic effects on bone healing [11]. Our recent study indicated that adding GSB to the composites comprising genipin-cross-linked gelatin and *β*-tricalcium phosphate enhances bone repair [7]. However, drug-drug interactions are inevitable owing to complicated components in GSB. Therefore, some investigators studied the therapeutic functions of pure active ingredient of GSB [13, 17].

Naringin, a polymethoxylated flavonoid and the main effective component of GSB, is commonly found in citrus fruits and many Chinese herbs [18, 19]. The potential benefits of naringin on bone metabolism and osteogenic responses have received considerable attention recently [17, 20]. For example, Wei et al. demonstrated that naringin could protect against retinoic acid-induced osteoporosis in Sprague Dawley rats [21]. Mandadi et al. indicated that naringin improves bone quality in orchidectomized male rats [22]. Pang et al. also revealed that naringin could protect against ovariectomized-induced bone loss in mice [23]. Zhou et al. revealed that naringin administration markedly promotes local bone formation and net bone growth under the conditions of titanium particle-induced osteolysis in a diabetic murine clavarial model [24].

To our knowledge, *in vivo* bone formation using a gelatin-*β*-tricalcium phosphate composite with naringin has never been investigated. In this study, the effects of various concentrations of naringin on the activity of bone cells were evaluated using the colorimetric assay, alkaline phosphatase (ALP) and tartrate-resistant acid phosphatase (TRAP) activity assays, and quantification of bone nodules. A porous biodegradable composite (GGT) containing genipin cross-linked gelatin and *β*-tricalcium phosphate was fabricated by the salt-leaching method to carry naringin (GGTN). The effect of naringin on the regeneration of defective bone tissue was examined by using GGTN as bone defect fillers in rabbit calvarial. The radiographic and histological features of the implants were evaluated as well.

## 2. Materials and Methods

### 2.1. Osteoblast Culture and Mixed Osteoblast/Osteoclast Coculture Systems

The biological effects of naringin on osteoblasts were evaluated using osteoblast-like MG-63 human osteosarcoma cells (BCRC number 60279, Food Industry Research and Development Institute, Taiwan). Cells were cultured in Dulbecco's modified Eagle's medium (DMEM; Gibco, Grand Island, NY) supplemented with 10% fetal bovine serum and 1% penicillin/streptomycin (Gibco) at 37°C under 5% CO_2_ in air. The medium was refreshed every 2 days. The adherent cells were allowed to reach ~80% confluence. The cells were then passaged in the culture, and cells at their second passage were used in the following experiments. 5 × 10^3^ cells/well of cultured MG-63 cells were seeded in individual wells of a 96-well tissue culture plate. After culturing for 24 hours, the culture medium was replaced with mixed solutions of a new culture medium and various concentrations of naringin (Sigma, St. Louis, MO) solutions in a volume ratio of 9 : 1 [25]. In the control group, the culture medium was mixed with phosphate-buffered saline (PBS) in a ratio of 9 : 1 for cell cultures. After culturing for 2 days, the proliferation and differentiation of MG-63 cells were determined by 3-(4,5-dimethylthiazol-2-yl)-2,5-diphenyl tetrazolium bromide (MTT; USB, Amersham Life Science, Cleveland, OH) assay and intracellular total ALP activity assay, respectively.

The effects of naringin on mixed bone cells were evaluated using the rat calvarial osteoprogenitor-splenic mononuclear cell coculture system [7, 26, 27]. Rat osteoprogenitor cells and splenic mononuclear cells were prepared from newborn (<3 days old) Sprague Dawley rats (obtained from National Laboratory Animal Center, Taiwan). Before the study, the ethical committee for animal experiments at the Central Taiwan University of Science and Technology, Taichung, Taiwan, approved the protocols. The calvarias of rat were isolated, stripped of soft tissues, washed three times with PBS, and digested in collagenase solution (Sigma) for 2 hours. The isolated cells were pooled, washed, and resuspended in a tissue culture medium [16]. Next, 1 × 10^2^ cells/well of osteoprogenitor cells and 5 × 10^3^ cells/well of splenic mononuclear cells in a ratio of 1 : 50 were cocultured in individual wells of a 96-well tissue culture plate for 6 days in a previous tissue culture medium with the addition of 10 nM 1*α*, 25-dihydroxyvitamin D_3_ [1*α*, 25(OH)_2_D_3_] (Sigma). The culture medium was refreshed every 2 days. After coculturing, the culture medium was replaced with mixed solutions of new culture medium and various concentrations of naringin solutions in a volume ratio of 9 : 1. In the control group, the culture medium was mixed with PBS in a ratio of 9 : 1 for cell cultures. After culturing for 2 days, the proliferation of bone cells from their precursor cells was determined by MTT assay.

### 2.2. MTT Assay for Cell Viability

After 2 days of culturing, the medium was replaced with 10 *μ*L/well of MTT solution (5 mg/mL) and 100 *μ*L/well of culture medium and incubated at 37°C for 4 hours to enable formation of insoluble dark-blue formazan crystals. The solution was then removed, and 100 *μ*L/well of acidic isopropyl alcohol (0.04 M of HCl in isopropyl alcohol) was added to all wells and mixed thoroughly to dissolve the crystals. After shaking for a few min, the optical density was measured using an enzyme-linked immunosorbent assay (ELISA) reader (uQuant; Bio-Tek Instruments Inc., Sunnyvale, CA) at a test wavelength of 570 nm against a reference wavelength of 650 nm. Experiments were carried out in three cultures.

### 2.3. Analysis of Alkaline Phosphatase for Osteoblast Differentiation

After 2 days of culturing, the medium was replaced with 20 *μ*L/well of 0.1% Triton X-100 (Sigma) and incubated at room temperature for 5 min for lysis of the cells. 100 *μ*L/well of a commercial ALP assay kit (Procedure no. DG1245-K, Sigma) was then added within 1 min. The kinetics of enzyme were elucidated using an ELISA reader, which measured the change in absorbance every minute for 15 min at 405 nm caused by p-nitrophenol production. The ALP activity was calculated using the slope of absorbance versus time [2]. Each experimental condition was repeated three times.

### 2.4. Quantification of Bone Nodules

Von Kossa's stain was utilized to examine the formation of the mineralized matrix. The number of mineralized nodules was counted [28]. Briefly, 2 × 10^4^ cells/well of cultured MG-63 cells were added to the culture medium containing 50 *μ*g/mL L-ascorbic acid (Sigma), 10 mM *β*-glycerophosphate (Sigma), and 10^−8 ^M dexamethasone (Sigma) in a 35 mm-culture dish. The culture medium was mixed with various concentrations of naringin solutions in a volume ratio of 9 : 1. In the control group, the culture medium was mixed with PBS in a ratio of 9 : 1 for cell cultures. The medium was refreshed every 3 days. After culturing for 21 days, cultures were washed with PBS three times and fixed in 2% glutaraldehyde (Acros) for 20 min. After a 20 min incubation with 5% silver nitrate (Union Chemical Works Ltd., Hsinchu, Taiwan) in the dark at room temperature, cells layers were rinsed three times in deionized water. The cells were exposed to ultraviolet light for 1 hour until color development was complete, and then the cells were treated with 5% sodium thiosulfate (Union Chemical Works Ltd.) for 2 min. Finally, cellular nodular structures were visualized by counterstaining with 0.1% nuclear fast red (Sigma) dissolved in 5% aluminum sulfate (JT Baker, Phillipsburg, NJ) for 5 min. Following rinsing in deionized water, the newly formed bone nodules were observed and counted with an optical microscope. Calcium mineral appears dark brown/black with this technique. Experiments were carried out in three cultures.

### 2.5. Analysis of TRAP for Osteoclast Differentiation

Several studies of mixed-bone cells culture system have indicated that the formation of mature osteoclasts requires 6 days [29]. The first 4 days is the period of the proliferation of osteoclast progenitors, and the final 2 days is the period of differentiation of osteoclasts. 2 × 10^2^ cells/well of osteoprogenitor cells and 1 × 10^4^ cells/well of splenic mononuclear cells in a ratio of 1 : 50 were added to a 48-well tissue culture plate to perform the TRAP test. The mixed-bone cells were cultured with various concentrations of naringin added at various periods. Next, naringin was added to the mixed-bone cells from the start of the culture to day 4 (the first period), from day 5 to day 6 (the second period), or from day 7 to day 8 (the third period). When naringin was added in the first or second period, mixed-bone cells were cultured for 6 days, while mixed-bone cells were cultured for 8 days when naringin was added in the third period. In the control group, the culture medium was mixed with PBS. The culture medium was replaced every 2 days with mixed solutions of a new culture medium and various concentrations of naringin solutions or PBS (control group) at a volume ratio of 9 : 1. 

TRAP activity was evaluated by measuring the amount of acid phosphatase (ACP) released from cells into the medium using a commercially available kit (Procedure no. 435, Sigma). Briefly, 30 *μ*L culture media was mixed with 100 *μ*L acid phosphatase reagent. The kinetics of enzyme were then measured using an ELISA reader, which monitored the change in absorbance every min for 5 min at 405 nm caused by p-nitrophenol production. The slope of absorbance versus time was directly proportional to the TRAP activity [9]. The TRAP activity test was performed on four replicate samples for each condition.

### 2.6. Preparation of Porous GGT and GGTN Composites

The GGT composites were prepared as described elsewhere [3, 9]. Briefly, a homogeneous 18 wt% gelatin solution was obtained by dissolving porcine gelatin (Bloom number 300, Sigma) in deionized water in a water bath at 80°C. While the gelatin solution cooled to 40°C, genipin solution (Challenge Bioproducts, Yunlin, Taiwan) at a concentration of 0.5 wt% was added to the gelatin solution to cause a cross-linking reaction at a constant temperature. After the solution was stirred for 2 min, *β*-tricalcium phosphate ceramic particles (Merck, Darmstadt, Germany) with grain sizes of 200–300 *μ*m and sieved sodium chloride particles of size 250–470 *μ*m were mixed with the gelatin-genipin mixture. The sodium chloride particles were dried in an oven at 170°C for 4 hours before use. The ratio of the weight of gelatin to tricalcium phosphate and that of salt particulates to gelatin/tricalcium phosphate/genipin composite were 1 : 3 and 3 : 1, respectively. Following vigorous stirring, the mixtures became increasingly viscous. They were transferred to plastic dishes to solidify and then frozen at −80°C for 30 min. The solidified mixtures were cut and shaped into cylindrical specimens of a particular size. The samples were then freeze-dried for 24 hours. The salt was leached out completely by immersing the samples in deionized water. Finally, the samples were frozen at −80°C for 24 hours and freeze-dried for another 24 hours to produce porous GGT composites. The dried cylindrical composites had a diameter of 10 mm and a thickness of 2 mm. The GGTN composites were prepared by a similar procedure to that used to prepare the GGT composites. A homogeneous 18 wt% gelatin solution was obtained by dissolving porcine gelatin in 10 mg/mL naringin solution, the optimal concentration determined from the results of the osteoblast culture and the mixed-bone cells culture. All samples were sterilized using *γ*-rays before use.

### 2.7. Biological Response of Rabbit Calvarial Bone

Experimental cranial implantation was conducted on twelve mature New Zealand white rabbits (2.5–3.0 kg, National Laboratory Animal Center, Taiwan). Rabbits were anaesthetized with intramuscular injection of Zoletil 50 (Virbac, France) and 2% Rompun solution (Bayer, Germany) (1 : 2 ratio, 1 mL/kg). The head of each rabbit was shaved and disinfected with 10% povidone-iodine solution (Chou Jen Pharmaceutical Co., Nantou, Taiwan). The cranial surface was exposed by making a midline incision, and the overlying parietal periosteum was then excised. Next, a full-thickness circular defect of the parietal bone with a diameter of 10 mm was produced using a drilling burr on a slow-speed dental handpiece that had been supplemented with 0.9% physiological saline without violating the dural or superior sagittal sinus. Two calvarial bone defects were created in each rabbit. One defect was filled with the sterile GGT composite (control group), and the other was filled with the sterile GGTN composite (experimental group) to evaluate their osteogenerative characteristics. Each composite sample was easily molded to the calvarial bone defect and did not require any fixation.

The repair of bone defect was evaluated radiographically and histologically. Anesthetized animals were sacrificed by administering an overdose of sodium pentobarbital at 2, 4, and 8 weeks post operatively, respectively. Four rabbits were examined at each time point. The craniectomy sites with 2-3 mm of contiguous bone were removed from each skull. Specimens were fixed in 10% phosphate-buffered formalin solution (Merck, Whitehouse Station, NJ) for 24 hours. They were then radiographed in a cabinet X-ray machine (MGU 100A, TOSHIBA Co., Japan) with a high contrast X-ray film at 22 keV, 10 mA for 40 s. New bone was revealed by the radiographic appearance of a calcified mass. For histological analysis, specimens were subsequently decalcified in a commercial medium (TBD-1 Rapid Decalcifier, Thermo Shandon, Pittsburgh, PA) for 48 hours. The specimens were dehydrated in a graded series of ethanol, immersed in xylene, and embedded in paraffin wax (Merck). They were then sectioned to 10 *μ*m thickness. Finally, sections were stained with hematoxylin and eosin (H and E; Sigma) to view histologically bone formation at the defect under an inverted optical microscope. 

### 2.8. Statistical Analysis

Numerical data were presented as mean ± standard derivation. Statistical analysis was performed using one-way analysis of variance (ANOVA) followed by* post hoc* Fisher's LSD multiple comparison test. The levels of statistical significance were set to *P* < 0.05.

## 3. Results

### 3.1. Effects of Naringin Concentration on Osteoblast

**Figure 1:**
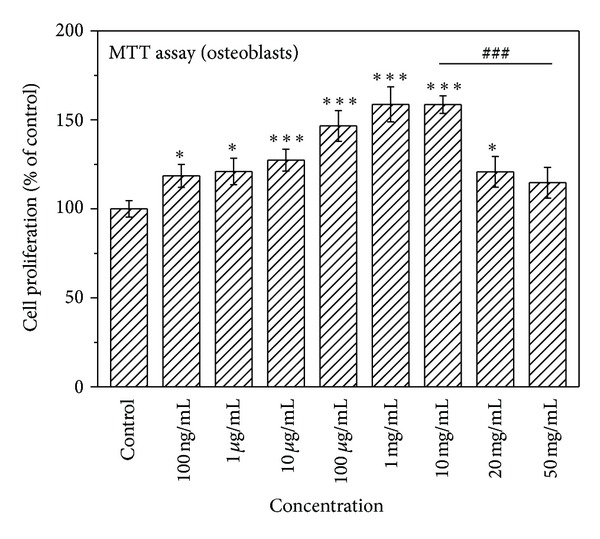
Effect of naringin on the proliferation of osteoblasts in the osteoblasts culture system, as determined by MTT assay. Results are expressed as percentage of control (*P < 0.05 and ***P < 0.001 versus control; ^###^ *P* < 0.001 versus 10 mg/mL).

Figure 1 illustrates the simulative effect of various concentrations of naringin on the proliferation of osteoblasts measured by MTT assay. Treatment with naringin at the concentrations between 100 ng/mL and 20 mg/mL significantly promoted the proliferation of osteoblasts (*P* < 0.05). In particular, 1 and 10 mg/mL of naringin increased the number of osteoblastic cells by 60%. However, the number of osteoblasts significantly decreased when the concentration of naringin was >10 mg/mL (*P* < 0.001). No statistically significant difference in the proliferation of osteoblasts was observed from that of the control group at 50 mg/mL. Therefore, higher concentrations than 10 mg/mL were not evaluated in the following study. 

**Figure 2:**
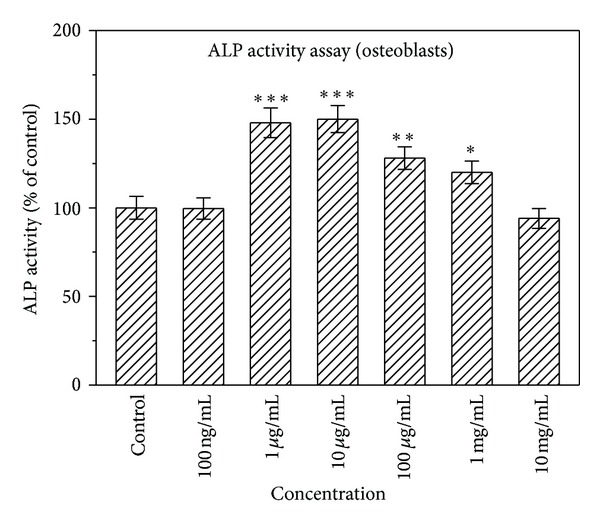
Effect of naringin on the differentiation of osteoblasts in the osteoblasts culture system using ALP activity assay. Results are expressed as percentage of control (*P < 0.05, **P < 0.01, and ***P < 0.001 versus control).

ALP, a membrane-bound enzyme, is a differentiation marker of early osteoblasts. Differentiated osteoblasts exhibited elevated ALP activity.  Figure 2 displays the effect of various concentrations of naringin on the ALP activity of osteoblasts. Compared with control, the ALP activity significantly increased when the concentrations of naringin ranged from 1 *μ*g/mL to 1 mg/mL (*P* < 0.05). In particular, low concentrations of naringin (1 and 10 *μ*g/mL) significantly increased osteoblastic cell differentiation by 50%. No statistically significant difference in the ALP activity was observed from that of the control group at 100 ng/mL and 10 mg/mL. 

**Figure 3:**
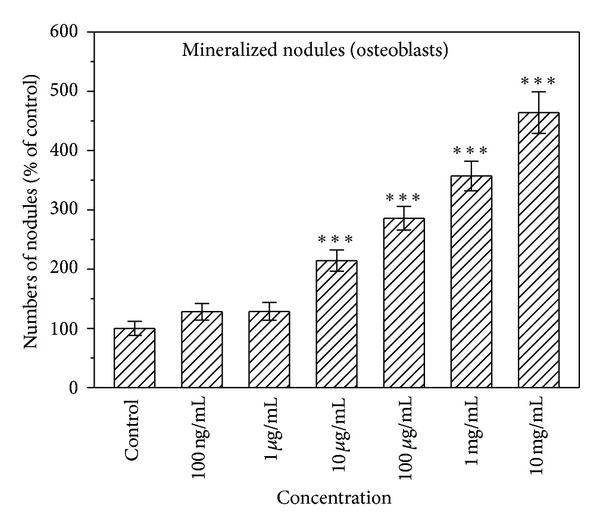
Effect of naringin on the numbers of total calcified nodules formed in the osteoblasts culture system, as determined by von Kossa's stain. Results are expressed as percentage of control (***P < 0.001 versus control).

Figure 3 shows the number of total calcified nodules formed in osteoblast cultures with different concentrations of naringin. Adding naringin to the cell cultures every 3 days significantly increased the number of total nodules formed, especially at a concentration of 10 mg/mL (*P* < 0.001). 

### 3.2. Effects of Naringin Concentration on Osteoclast

**Figure 4:**
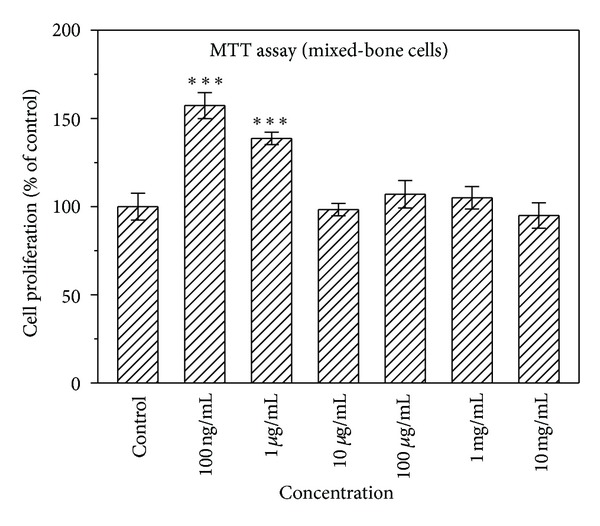
Effect of naringin on the proliferation of mixed-bone cells in the osteoblast/osteoclast coculture system, as determined by MTT assay. Results are expressed as percentage of control (***P < 0.001 versus control).

A coculture system was adopted to evaluate the effects of naringin concentration on the proliferation of osteoclast progenitor cells and on the differentiation of osteoclasts and mature osteoclasts. The conditions of the coculture system were close to the physiological conditions of bone.  Figure 4 displays the effect of naringin on the proliferation of mixed-bone cells assessed by MTT assay. When mixed-bone cells were cultured with low concentrations of naringin, 100 ng/mL and 1 *μ*g/mL, for 2 days, the proliferation of mixed-bone cells was significantly increased compared to control (*P* < 0.001). When the concentration of naringin was >10 *μ*g/mL, the number of mixed-bone cells did not increase significantly. 

**Figure 5:**
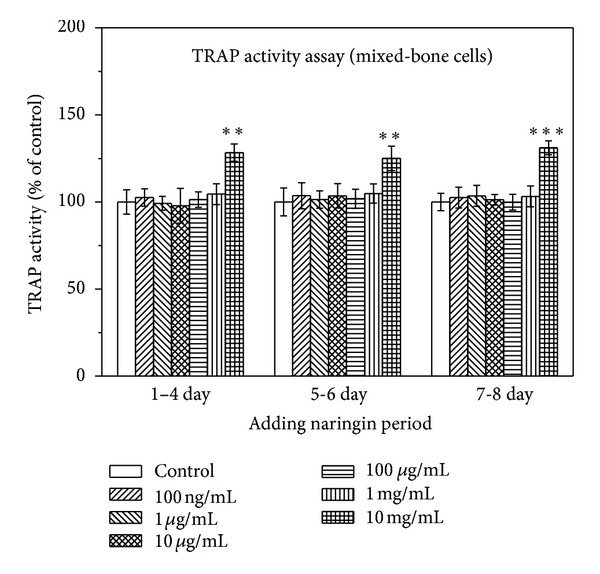
Effect of naringin on the TRAP activity of the mixed-bone cells, after naringin was added in three periods. Results are expressed as percentage of control (**P < 0.01 and ***P < 0.001 versus control).

Figure 5 shows the results of TRAP activity assay for cultured mixed-bone cells with various concentrations of naringin added after different periods. Naringin at concentrations lower than 1 mg/mL did not affect the TRAP activity of mixed-bone cells. However, naringin at concentration of 10 mg/mL had significant effect on the TRAP activity. When naringin was added to osteoclast progenitor cells during the first 4 days of the proliferative phase, 10 mg/mL of naringin markedly increased TRAP activity (*P* < 0.01). The increase is 28%. When naringin was added to osteoclast on day 5 or 6, 10 mg/mL of naringin significantly promoted TRAP activity (*P* < 0.01). The increase is 25%. As naringin was added to differentiated mature osteoclasts after day 7, 10 mg/mL of naringin significantly increased TRAP activity (*P* < 0.001). These results indicate that naringin at high concentration could enhance osteoclast progenitor cells and osteoclasts formation, which would reduce the number of splenic mononuclear cells. Therefore, it may result that the total number of cells cultured with high concentration of naringin did not increase significantly compared to control. 

In the osteoblast culture, the most effective concentration of naringin on the proliferation of osteoblasts and nodule formation was 10 mg/mL. The most effective concentrations of naringin in terms of the differentiation of osteoblasts were 1 and 10 *μ*g/mL. In the mixed-bone cells culture, the most effective concentrations of naringin on the proliferation of mixed-bone cells were 100 ng/mL and 1 *μ*g/mL. TRAP activity assay revealed that 10 mg/mL naringin enhances the proliferation of osteoclast progenitor cells and the differentiation of osteoclasts and mature osteoclasts. Additionally, naringin at concentration of 10 mg/mL did not significantly affect the differentiation of osteoblasts and the proliferation of mixed-bone cells. Thus, 10 mg/mL of naringin was added to the GGT composites for use in subsequent animal implantation experiments.

### 3.3. **In Vivo** Evaluation of GGT and GGTN Composites

A GGTN composite was fabricated using a 10 mg/mL optimal concentration of naringin. GGTN and GGT composites were implanted into bony defects in the calvaria of rabbits. All animals survived throughout the experiment. No wound infection, scalp effusion, hematoma, festers, or other complications were observed at the implantation sites. Gross observation of the entire calvaria indicated that the composite is intimately incorporated into the surrounding host bone. 

**Figure 6:**
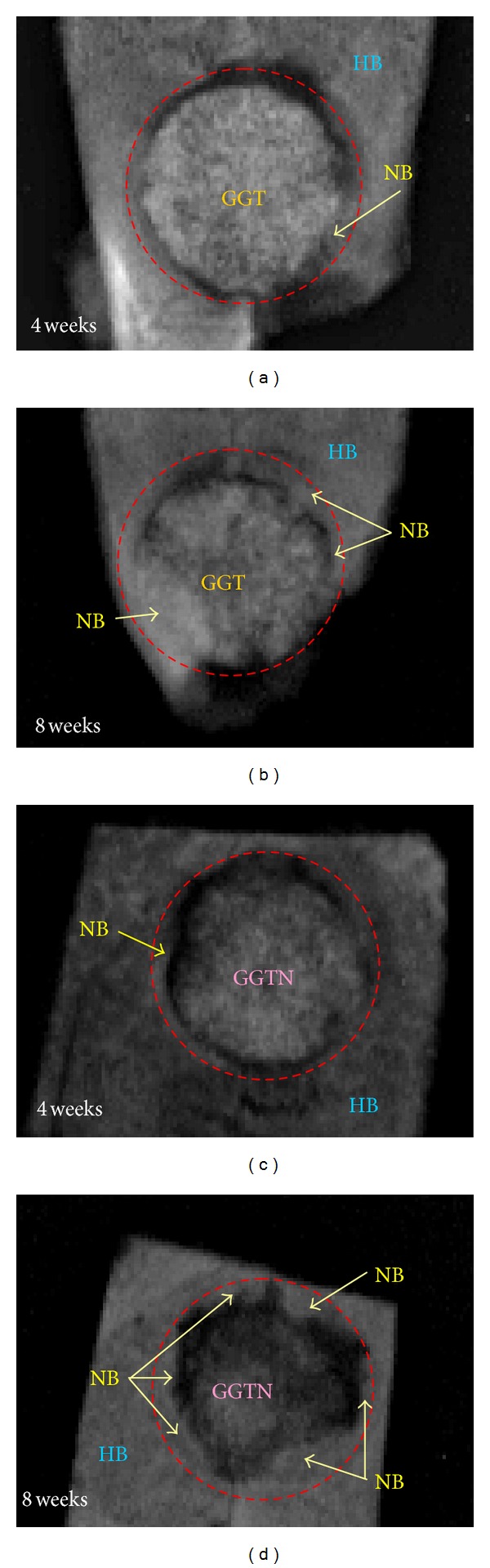
Radiographs of calvarial bone-covered implant removed after (a), (b) GGT composites and (c), (d) GGTN composites were implanted into the calvarial bone defects for (a), (c) 4 and (b), (d) 8 weeks (HB = host bone, NB = new bone, and dotted line indicates the original margin of the calvarial bone defect).

X-ray radiographs were obtained to determine whether the new osseous tissues are completely calcified new bone. The efficacy of both GGTN and GGT composites in the repair of the calvarial bone defects was evaluated to identify which material better enhanced healing.  Figure 6 shows radiographs of 10 mm skull defects in rabbits following the application of GGT and GGTN composites. At 4 weeks after surgery, new bone formed at the host bone-composite interface (Figures 6(a) and 6(c)). The newly formed bone partially replaced the composite, revealing a decrease in the volume of the composite. Additionally, the newly formed bone laid down from the margin toward the center of the calvarial bone defect, causing an irregular shape of the rounded bone defect. Furthermore, the area of new bone formed using the GGTN composite exceeded that formed using the GGT composite (Figure 6(a)).

At 8 weeks after surgery, the area of the calvarial bone defect was markedly smaller than that observed in the 4-week radiograph, indicating that the area of new bone increased over time (Figures 6(b) and 6(d)). Moreover, this figure revealed a greater amount of new bone ingrowth in the GGTN composite at the same time of implantation, suggesting that the release of naringin from the degraded GGTN composite promoted new bone growth. Additionally, serial postoperative radiographs demonstrated that the GGTN and GGT composites tended to biodegrade with time. This degradation was accompanied by the deposition of new bone. Moreover, the defect repaired with GGTN composite exhibited nearly complete radiopacity across the interface between the host bone and the composite (Figure 6(d)). This finding suggests that the rate of biodegradation of the GGTN composite closely matched the rate of generation of new bone. Therefore, the GGTN composite was not obviously separate from the adjacent host bone tissue. 

**Figure 7:**
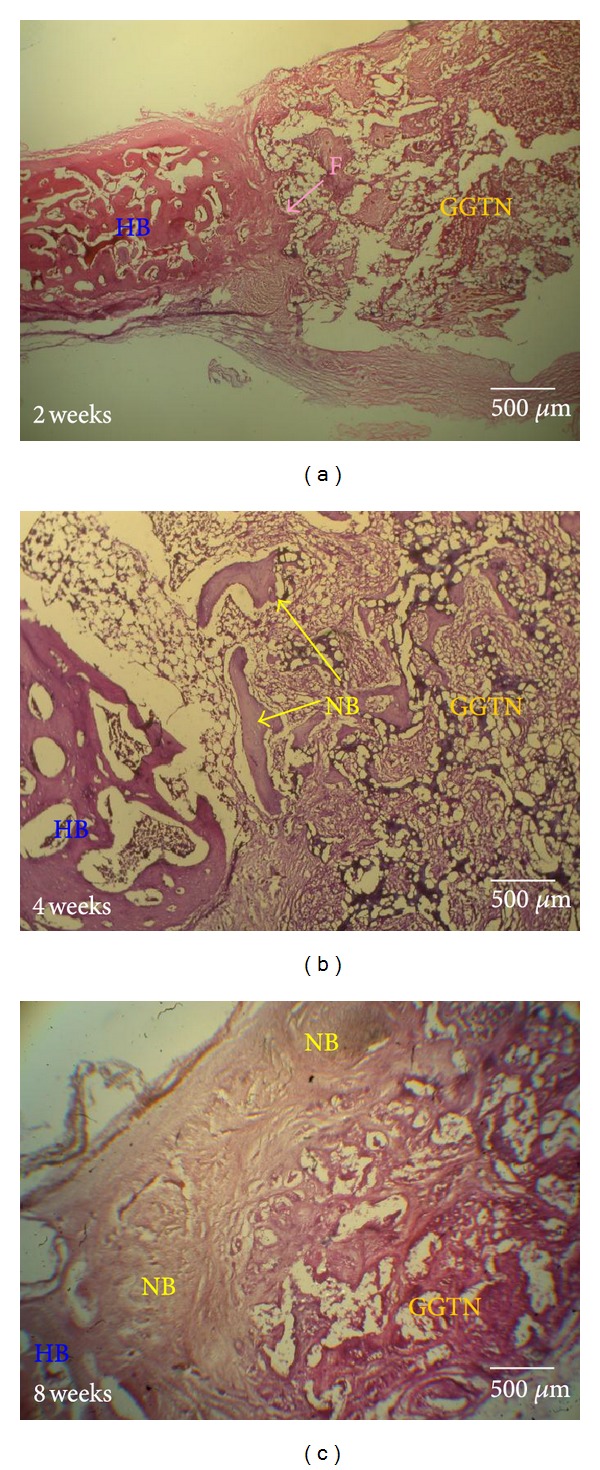
Histological images of H and E-stained GGTN composites implanted in calvarial defects for (a) 2, (b) 4, and (c) 8 weeks (F = fibrous connective tissue, HB = host bone, and NB = new bone).

Histological examination was performed to evaluate the progress of damaged bone restoration using the GGTN composite. Following harvest, the composite was stained with H and E. Two weeks after the operation, the histological observation of the calvarial bone defect did not reveal new bone regeneration (Figure 7(a)). Only a bridge of fibrous connective tissue was observed across the bone defect. After 4 weeks of recovery, the new bone began to replace the GGTN composite (Figure 7(b)). The osteoconductive process of the calvarial host bone was directed toward the bone defect, forming new bone near the host bone-composite interface. After 8 weeks, the bone regeneration was accelerated (Figure 7(c)). More new bone extended centripetally from the calvarial margins. The histological finding was consistent with the radiographic finding. As the GGTN composite biodegraded and released stimulatory factors, new bone growth into the composite increased. At 8 weeks after implantation, new bone had replaced a significant amount of the GGTN composite.

## 4. Discussion

As an important biological process for renewal and repair of damaged bone, bone remodeling is initiated by osteoclastic bone resorption and subsequent new bone formation by osteoblasts. The balance between bone resorption and bone formation is necessary to maintain skeletal integrity and normal bone functions. Previous studies have demonstrated that naringin, a polymethoxylated flavonoid found in citrus fruits and many Chinese herbs, significantly affects bone cell activity. For instance, Wong and Rabie reported that naringin significantly increases proliferation and total protein content of rat osteoblast-like UMR-106 cells [30]. The highest concentration (100 nM) has the greatest effect. However, naringin does not alter ALP activity of UMR-106 cells at 2 days and mildly increased it at 3 days at high concentration (100 nM). Wang et al. found that naringin has a does-dependent effect on promoting ALP activity of UMR-106 cells and significantly increased the ALP activity at most of the concentrations of 100 nM and 1000 nM [31]. However, naringin does not show effects on the proliferation of UMR-106 cells at the tested concentrations (0.1–1000 nM). Wu et al. discovered that 3 *μ*M of naringin induces the proliferation, differentiation, and maturation of murine osteoblastic MC3T3-E1 cells, as well as increases bone morphogenetic protein-2 (BMP-2) expression in osteoblasts [20]. They also found that BMP-2 is a target protein for naringin signaling pathway. Zhang et al. revealed that 1–100 *μ*g/mL of naringin promote the proliferation and ALP activity of human bone mesenchymal stem cell, as well as increase the quality and quantity of calcium node formation [32]. The 100 *μ*g/mL group shows the most significant difference. These studies demonstrated that naringin has different concentration-dependent effects on the proliferation and differentiation of different osteoblastic cell lines. Moreover, the effects of various concentrations of naringin on the activities of mixed-bone cells were not evaluated in these studies. 

The osteosarcoma cell line MG-63 has been commonly used as a model cell line for elucidating osteogenic cell behavior on biomaterials because of its high proliferative capacity and its potential for differentiation and mineralization. This study demonstrated that naringin markedly enhances the proliferation and differentiation of MG-63 cells and formation of nodule. Additionally, naringin has concentration-dependent effects on the proliferation and differentiation of MG-63 cells and nodule formation. Naringin at 10 mg/mL significantly accelerates osteoblastic proliferation and nodule formation in the osteoblast culture and enhances the proliferation of osteoclast progenitor cells and the differentiation of osteoclasts and mature osteoclasts in the mixed-bone cells culture. Additionally, naringin at 10 mg/mL does not affect the ALP activity of osteoblasts and the mitochondrial activity of mixed-bone cells. Therefore, 10 mg/mL of naringin is the most effective concentration for the activity of bone cells.

Our previous studies developed a biodegradable GGT composite composed of genipin-cross-linked gelatin and *β*-tricalcium phosphates as a bone substitute. The composite can support the proliferation and differentiation of osteoblasts [2]. Additionally, the GGT composite exhibits excellent osteoconduction *in vivo *[4]. An ideal bone substitute should have characteristics of osteoconduction and osteoinduction. However, the GGT composite is not osteoinductive. Adding a bone-inducing agent favorably accelerates the ingrowth of new bone into a defect site. The traditional Chinese medicine GSB promotes bone repair and is highly promising as a bone-inducing agent. As is generally recognized, naringin is the main active component of GSB. In this study, the optimal amount of naringin (i.e., 10 mg/mL) was mixed with the GGT composite (GGTN) to promote bone regeneration further.

This study also investigated the* in vivo* activity of naringin in a rabbit calvarial defect model. Many researchers have demonstrated that the trephine skull defects larger than 8 mm in diameter can heal spontaneously only by the invasion of soft tissue in the period selected in this study (2, 4, and 8 weeks) and not by bony bridging. Such defects are reliable delayed-healing models [33]. Therefore, the calvarial defect model is an extremely valuable means of evaluating the bone-regenerative capacity of the porous GGTN and GGT composites. Gross examinations revealed that the composites are perfectly molded into the bone defects, remaining in place during the entire postoperative period. As the composites degraded, some of their components released into the defects, such as gelatin molecules and calcium ions, were considered to be nutrients for new bone formation [2]. Naringin and unchained genipin, used as a natural cross-linker, were also released from the GGTN composite. The brain tissues beneath the GGTN composite did not exhibit any cortical inflammation or scar formation, indicating that naringin and unchained genipin do not harm the surrounding bone tissues.

Radiographic analysis revealed new bone growth into the calvarial defects in the porous GGT and GGTN composites. It also confirmed progressive wound healing, implant resorption, and infiltration of new bone into the implant construct over time. The area of the bone newly formed using the GGTN composite exceeded that formed using the GGT composite. Additionally, examination of the H and E-stained sections of the craniectomy sites revealed that new bone replaced a significant amount of the GGTN composite. Naringin was gradually released from the biodegradable composite and was considered to affect new bone formation positively. According to the cell culture test, naringin can promote the proliferation of osteoclast progenitor cells, the differentiation of osteoclasts and mature osteoclasts, and the proliferation of osteoblasts. Therefore, naringin not only could trigger the osteoclasts to resorb bone debris, but also stimulate the osteoblasts to form new bone. This observation is consistent with the findings of previous studies. Wong and Rabie reported that naringin in collagen matrix enhances new bone formation locally [34]. The amount of new bone produced by naringin in collagen matrix was significantly more than that produced by autogenous endochondral bone and collagen matrix alone. Therefore, naringin can stimulate bone formation *in vivo*. Moreover, above studies suggest that naringin can promote new bone formation by providing an effective biodegradable delivery system.

In conclusion, the present study demonstrated that naringin has beneficial effects on the bone cells culture. Moreover, naringin mixed with GGT composite successfully enhanced bone regeneration with good osteoconductive activity. Therefore, incorporating naringin to a porous GGT composite makes it highly promising for bone repair. Nevertheless, our research is only a preliminary study to investigate the feasibility of using naringin to enhance bone regeneration. Further studies, such as bone biochemical marker assays, are needed to define the role of naringin in bone repair *in vivo*.
